# Leaf-Counting in Monocot Plants Using Deep Regression Models

**DOI:** 10.3390/s23041890

**Published:** 2023-02-08

**Authors:** Xinyan Xie, Yufeng Ge, Harkamal Walia, Jinliang Yang, Hongfeng Yu

**Affiliations:** 1 School of Computing, University of Nebraska-Lincoln, Lincoln, NE 68588, USA; 2 Department of Biological Systems Engineering, University of Nebraska-Lincoln, Lincoln, NE 68583, USA; 3 Department of Agronomy and Horticulture, University of Nebraska-Lincoln, Lincoln, NE 68583, USA

**Keywords:** plant phenotyping, leaf-counting, convolutional neural network, regression, Grad-CAM

## Abstract

Leaf numbers are vital in estimating the yield of crops. Traditional manual leaf-counting is tedious, costly, and an enormous job. Recent convolutional neural network-based approaches achieve promising results for rosette plants. However, there is a lack of effective solutions to tackle leaf counting for monocot plants, such as sorghum and maize. The existing approaches often require substantial training datasets and annotations, thus incurring significant overheads for labeling. Moreover, these approaches can easily fail when leaf structures are occluded in images. To address these issues, we present a new deep neural network-based method that does not require any effort to label leaf structures explicitly and achieves superior performance even with severe leaf occlusions in images. Our method extracts leaf skeletons to gain more topological information and applies augmentation to enhance structural variety in the original images. Then, we feed the combination of original images, derived skeletons, and augmentations into a regression model, transferred from Inception-Resnet-V2, for leaf-counting. We find that leaf tips are important in our regression model through an input modification method and a Grad-CAM method. The superiority of the proposed method is validated via comparison with the existing approaches conducted on a similar dataset. The results show that our method does not only improve the accuracy of leaf-counting, with overlaps and occlusions, but also lower the training cost, with fewer annotations compared to the previous state-of-the-art approaches.The robustness of the proposed method against the noise effect is also verified by removing the environmental noises during the image preprocessing and reducing the effect of the noises introduced by skeletonization, with satisfactory outcomes.

## 1. Introduction

To estimate the yield of crops, plant scientists study various chemical or physical traits. Among them, leaf numbers are a commonly employed trait and an important component of plant biomass, especially in the vegetative stages. We use the number of leaves to determine the growth stages of plants for sorghum and maize. The two most important physiological functions of plants, photosynthesis and transpiration, occur in plant leaves and are hence closely associated with leaf number. Traditional methods of manually collecting measures are laborious and error-prone, and may damage plants. With the advancements in machine learning, various image-based approaches have been developed to investigate plant properties while preventing damage to plants and lowering costs. These approaches can be roughly divided into two categories. The first category is to segment individual leaves from the image of a plant and then count the leaf number. The second category is to teach regression models to count leaves from images directly. Both categories of solutions [1,2,3] achieve appropriate accuracy for rosette plants [4,5], where a plant has a circular arrangement of leaves around the stem and leaves are relatively easy to perceive from top-view images related to the plant.

However, there is a lack of effective solutions to tackle leaf-counting for monocot plants (e.g., sorghum and maize), which have elongated stalkless leaves with parallel veins. Compared to rosette plants, monocotyledon leaves have more flexibility in shapes and sizes. They grow roughly symmetrically, aligning with stems, and can easily incur occlusions and overlaps among them. It is often impossible to perceive all leaves of a monocot plant from a single view, particularly at its later growth stages. Most existing works required a large-scale dataset and usually failed to count the number of leaves with overlaps or occlusions. Jiang et al. [6] used a regression-based approach with Google Inception Net V3 [7] associated with Fisher vector coding [8] to count the number of leaves in the maize plant. The absolute counting difference for the testing dataset was 0.35, with a mean squared error (MSE) of 0.31. The misclassified images were mainly from leaves near the pots, overlapped leaves, and self-occluded leaves. Miao et al. [9] used the Resnet18 model [10] as their regression-based counting method, and the fast R-CNN model [11] as their segmentation-based method. The best result was achieved with an accuracy of 0.56, when all leaf tips were visibly captured. They used more than 15,000 maize and sorghum images and annotated the leaf counts and the bounding boxes for each leaf tip on all the images. Other researchers attempted to improve leaf-counting by applying 3D reconstruction of a plant from the multiple perspectives of 2D images (e.g., [12]). However, depth information was required during the construction process, which was not always available for many plant phenotyping systems.

In this paper, we develop a regression-based approach for counting leaves in monocot plants. We design our regression model based on Inception-Resnet-V2 [13]. Moreover, we enhance the input to the regression model by extracting leaf skeletons to gain more topological information and applying augmentation to enhance the structural variety of the original images. We believe that skeleton-structured images can help the model find commonalities among the structures of monocot plants and generalize the model to solve the leaf-counting problem. In addition, the augmentation methods, such as rotation and flipping, can expand the varieties and the densities of plant shapes for model training. We train and validate models with sorghum datasets, and evaluate them by predicting the number of leaves in both sorghum and maize plants. In these experiments, 1500 sorghum images and 150 maize images are employed and annotated with their corresponding numbers of leaves. Finally, we investigate the leaf spatial features that significantly contribute to the regression results of the total leaf number in sorghum images.

## 2. Materials and Methods

### 2.1. Overview

In this work, we advocate regression-based approaches because they require marginal efforts to annotate leaf numbers and can significantly reduce the costly and time-consuming labeling process. However, the existing regression-based approaches cannot fully address monocot plants due to leaf occlusion problems, unclear plant structures resulting from the illumination, and a lack of advanced models [6,9]. To address these issues, we note that a combination of advanced architecture, a distinct plant structure representation, and a wide variety of leaf shapes is promising in improving the accuracy of regression-based approaches for counting the leaves of monocot plants. Specifically, we observe that the Inception-ResNet-V2 architecture [13] integrates the advantage of the inherent significant connections from the ResNet network [10] and the high efficient layers from the Inception network [7], and thus is more suitable to be used as the backbone of our new network architecture. To overcome the problems of ambiguous leaf structures and occluded leaves, we extract skeleton structures from original plant images and use skeletons as a new form of data due to their intuitive representations of the object shapes and the inner connection relationships [14]. To obtain competitive performances with the existing works, we employ augmentation methods to achieve a similar level of dataset size. We apply the geometrical transformations [15,16] to all training datasets so that they can be largely expanded, and the varieties of leaf shapes are remarkably increased.

We introduce our network architecture in Section 2.2. Before they are input into our network, all original images are preprocessed to extract and resize the portions of plants, which will be described in Section 2.3. We also introduce the skeletonization process in Section 2.4 and the augmentation process in Section 2.5. We describe dataset sources in Section 2.6. Then, we show implementation details in Section 2.7, including implementation tools and hyper-parameters. In Section 2.8, we investigate the performance of models on these images using several evaluation metrics.

### 2.2. Network Architecture

**Figure 1:**
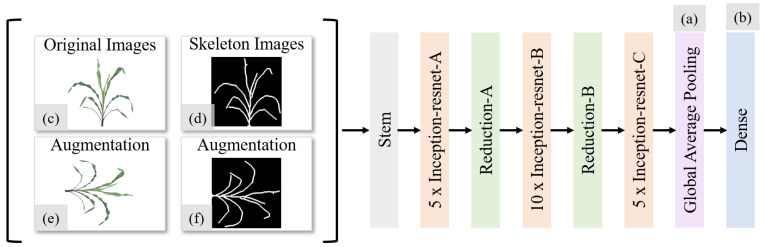
Our network architecture based on Inception-ResNet-v2 with main modifications: (**a**) global average pooling layer and (**b**) dense layer with one unit. The input to the network includes a combination of original images (**c**), skeleton images (**d**), and their augmentations (**e**,**f**).

Deep neural networks (DNN) have shown superior performance when they are widely applied to multiple fields [17,18,19,20,21,22]. Figure 1 shows the architecture of our DNN. We employ the Inception-ResNet-V2 architecture [13] as the backbone to predict the number of leaves in monocot plants. This architecture contains remarkable deep Inception networks [7], which significantly scale up networks and are employed in many applications, such as multimedia classification [23], visual saliency prediction [24], disease detection [25,26], and concrete crack-detection [27], etc. Our architecture also replaces the filter concatenation stage of the Inception architecture with residual connections [10,28] that can address the degradation problem when the model starts to converge, as shown in the orange boxes in Figure 1. The original Inception-ResNet-v2 model is trained on a 1000-class ImageNet dataset [29] to classify the object into 1 of 1000 categories. The input size of the original architecture is (299×299×3), and the output of the original architecture is 1 of 1000 classes. It has been used to predict the number of leaves in rosette plants [30] and classify rice leaf diseases [31]; both showed superior performances.

The original Inception-ResNet-V2 uses a softmax layer to predict the probability of each category in a classification problem. The objective of our network is to predict the number of leaves in monocot plants. We replace the softmax layer with one global average pooling layer (Figure 1a) and one dense layer (Figure 1b). The pooling layer has the potential to find significant features, which will be explored in Section 3.2. The dense layer only has one unit for building a regression model and outputs the predicted leaf-counting number.

Apart from the original plant images (Figure 1c), we convert an original plant image to a skeleton-structured image in order to obtain a more clear and more intuitive representation of the plant (Figure 1d), and the process is detailed in Section 2.4. In addition, we also apply augmentation methods to the original image (Figure 1e) and the skeleton image (Figure 1f) to extend the size and the diversity of the training datasets, and the process is detailed in Section 2.5. The combination of the original images, the skeleton images, and their augmentations will then be used as the inputs to our network. We examine the effect of different combinations in Section 3.

### 2.3. Image Preprocessing

**Figure 2:**
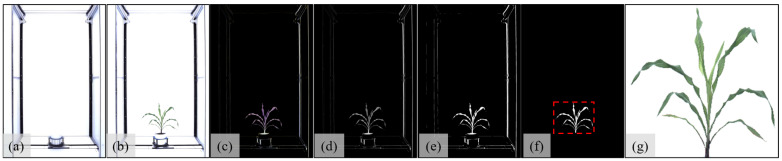
Image preprocessing with an example of a sorghum image: (**a**) average background image, (**b**) original image, (**c**) difference image, (**d**) difference image in gray-scale space, (**e**) binary image, (**f**) cropped binary image, and (**g**) cropped plant image from the original image.

We apply image preprocessing to extract and resize plant images from the original images. These steps can generate more concise images that only contain plants, have reduced sizes, which are suitable for our modified Inception-ResNet-V2 architecture. The entire image preprocessing process is demonstrated in detail as follows and also shown in Figure 2.

First, we remove the redundant equipment pixels in the background, as all the images have the same equipment which occupies substantial pixels as a background but is less relevant for leaf-counting. At first glance, this can be easily performed by subtracting the background equipment pixels from each image. However, these background pixels cannot be easily extracted from a single image because the equipment pixels of individual images have non-negligible illumination differences and possibly slight position differences. Thus, we randomly select 50 images and remove the whole plant from each image to generate 50 background images. Then, we average all background images and generate an average background image from them, as shown in Figure 2a, which can facilitate us to remove background pixels from each image. A difference image between an original plant image (Figure 2b) and averaged background image can roughly give us the pixels covered by the plant but possibly with certain noises (e.g., grass, bare soil, etc.), as shown in Figure 2c. We use connected components to remove these noises and get more accurate plant pixels. Specifically, we convert the difference image from the RGB space to the grayscale space (Figure 2d) and apply the Otsu binarization to obtain a binary image with fewer noises (Figure 2e). Then, we detect connected components in the binary image, and their geometric areas correspond to the plant pixels.

We use the largest connected region besides the background to create a bounding box of the plant pixels (Figure 2f) to crop the plant out from the original image. Each plant image is resized to the resolution of 299×299 used in our deep neural network (DNN) architecture, as shown in Figure 2g.

There are two resolutions, 2056×2454 and 6576×4384, among the original images. The preprocessing steps generate the images containing only plants and with the unified resolution 299×299 for the subsequent model training and experiments.

### 2.4. Image Skeleton

Jiang et al. [6] argued that the misclassified images in their work are mainly influenced by the leaf color change caused by the illumination. We also notice that the non-uniform leaf color can cause disconnections of a single leaf in an image, dividing one leaf into parts and confusing a model, causing it to make wrong predictions. To correct these misleading effects, the skeleton structures are considered. Du et al. [32] believed that the skeleton structure can provide the topology of the object shape and thus an intuitive representation. Gaillard et al. [14] separated the sorghum plant into leaves and stems based on the 3D plant skeletons, including endpoints identification, root identification, branch finding, and branch pruning. Their methods can generate reasonable skeleton structures for the sorghum images without occluded leaves but fail for ones with occluded leaves.

**Figure 3:**
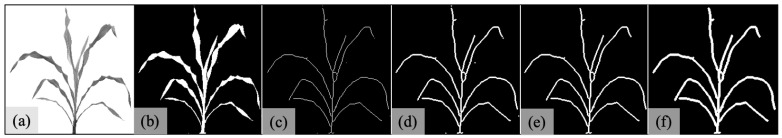
Skeleton structure steps: (**a**) gray-scale space image converted from Figure 2g; (**b**) binary image; (**c**) single-pixel wide skeleton image; (**d**) first dilation image; (**e**) small noises removed image; (**f**) second dilation image.

In our work, we extract the skeleton structures from the datasets to reduce color and shape differences, and then intuitively present the relationships among leaves. Figure 3 demonstrates the steps to generate skeleton structures from the cropped plant images (Figure 2g).

First, we convert the original cropped image (Figure 2g) to the grayscale space (Figure 3a), and apply the Otsu binarization to obtain a binary image (Figure 3b). The binary image is then thinned to a single-pixel-wide skeleton (Figure 3c) by removing pixels over the image to obtain a rough topology of the plant shape [33]. Then, we dilate the single-pixel wide skeleton to connect the discontinuous leaf segments, as shown in Figure 3d. After the first dilation, we consider the connected components with the corresponding areas smaller than a threshold to be the small noises and remove them from the first dilation images, as shown in Figure 3e. We empirically set the threshold as 50 pixels, which enables us to successfully remove noise pixels in practice. Finally, we dilate the denoised skeleton image again to enhance the skeleton structure, as shown in Figure 3f. After the second dilation operation, the binary image is employed as the final skeleton structure image and input to our modified Inception-ResNet-V2 architecture.

### 2.5. Image Augmentation

We also consider the augmentation methods in our work. A more extensive training dataset usually provides more useful features that can be learned during the training process, which can help reduce the overfitting problem and achieve a more accurate result for DNN architectures [34]. However, annotating massive images can be time-consuming and labor-intensive; for example, they may require multiple persons to work on thousands of images for several days. To solve this problem, we use data augmentation, a regularization scheme to artificially increase the dataset size and the example variety, preserve the same labels, and can effectively reduce the labeling cost [35]. Krizhevsky et al. [36] applied geometric transformations and color space augmentations on ImageNet [37] and achieved state-of-the-art results. Geometric transformations contain the operations such as flipping, color space, cropping, rotation, translation, and noise injection [38]. Due to their non-realistic or non-logical synthetic images, such transformations are generally not applicable to plants [39]. However, several geometric transformations maintaining the object’s natural properties are still applicable, to some extent. For example, Miao et al. [9] randomly horizontally flipped images; Abed et al. [15] and Zhang et al. [16] applied different rotation and flipping transformations to their leaf samples to artificially increase the number of images in the dataset to achieve better generalization. In our work, we use similar geometrical transformations to enlarge training datasets’ size and diversity. To investigate the effect of the non-realistic synthetic images on regression performance, we employ both realistic synthetic images and non-realistic synthetic images.

**Figure 4:**
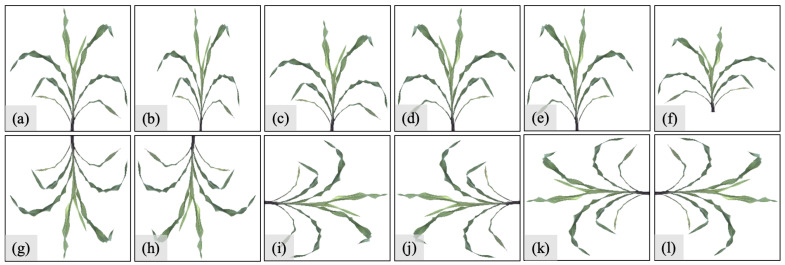
Image augmentation methods applied on (**a**) resized original image generated from image preprocessing: (**b**) compressed vertically; (**c**) compressed horizontally; (**d**) flipped vertically; (**e**) flipped and compressed vertically; (**f**) flipped vertically and compressed horizontally; (**g**) flipped horizontally; (**h**) rotated by 180 degrees; (**i**) rotated clockwise by 90 degrees; (**j**) rotated clockwise by 90 degrees and flipped vertically; (**k**) rotated counterclockwise by 90 degrees; and (**l**) rotated counterclockwise by 90 degrees, and then flipped vertically.

Figure 4 shows our image augmentation using a resized original image generated from our image preprocessing step as an example. Eleven transformation methods are used in our work, which can be divided into two types. The first row (Figure 4b–f) corresponds to the methods that do not produce non-logical synthetic images, and the second row (Figure 4g–l) includes the methods that produce unrealistic synthetic images:

- Original plant image with a size of 299×299×3 (Figure 4a);
- Vertically compress the original image (Figure 4b);
- Horizontally compress the original image (Figure 4c);
- Vertically flip the original image (Figure 4d);
- Vertically flip and compress the original image (Figure 4e);
- Vertically flip and horizontally compress the original image (Figure 4f);
- Horizontally flip the original image (Figure 4g);
- Rotate the original image by 180 degrees (Figure 4h);
- Clockwise rotate the original image by 90 degrees (Figure 4i);
- Clockwise rotate the original image by 90 degrees and then vertically flip it (Figure 4j);
- Counterclockwise rotate the original image by 90 degrees (Figure 4k);
- Counterclockwise rotate the original image by 90 degrees and then vertically flip it (Figure 4l).

After these transformations, if an image is smaller than 299×299×3, the white background is padded to the image to make it 299×299×3.

### 2.6. Dataset Description

We use both sorghum plant images and maize plant images to evaluate the model performance on general monocot plants. All images were taken at the University of Nebraska-Lincoln’s Greenhouse Innovation Center [40].

Our dataset includes images of 80 sorghum plants and 42 maize plants during their growth. The plants with tassel are not analyzed in our dataset; this mainly occurs after 50 DAP (days after planting) [41]. Therefore, all images were taken between 18 DAP and 50 DAP. For each plant, images were taken at every 36${}^{\circ }$ around the plant growth direction on each day. We manually selected our datasets from the images with the angle of 0${}^{\circ }$, 36${}^{\circ }$, 72${}^{\circ }$, 108${}^{\circ }$, and 144${}^{\circ }$ due to the similar shape and the same leaf-counting numbers from the 180${}^{\circ }$ rotation along the growth direction.

### 2.7. Implementation

We implemented our models in Python 3.9. The training processes were performed on NVIDIA GeForce RTX 3090 with the computing capability of 8.6, 10496 CUDA cores, and 24 GB off-chip global memory. The codes were compiled with the CUDA 11.2 compiler. All experiments in this work were conducted on our modified Inception-ResNet-v2 model on the ImageNet dataset. During the training process, all layer weights were allowed to be tuned. Our study mainly compares the performance among several datasets resulting from different augmentation methods and plant topologies. Therefore, we set the same hyperparameters for all experiments. Based on the existing leaf-counting studies [30,42,43] from computer vision problems in plant phenotyping (CVPPP) [5,44,45], the hyper-parameters used for training are listed in Table 1.

### 2.8. Evaluation Metrics

We modify the original Inception-ResNet-V2 architecture to a regression-based model, and predict the actual counted number of leaves in each plant image. We perform both quantitative and qualitative evaluations to analyze the model performance.

#### 2.8.1. Quantitative Evaluation Metrics

To quantitatively compare our results with the existing works using similar datasets, we employ several metrics, including RMSE (root mean squared error), $R^{2}$ (R2 coefficient of determination), and accuracy:

(1) $$RMSE=\sqrt{\frac{\sum_{i=1}^{n}{\left(y_{i}-{\hat{y}}_{i}\right)}^{2}}{n}},$$

(2) $$R^{2}=1-\frac{\sum_{i=1}^{n}{\left(y_{i}-{\hat{y}}_{i}\right)}^{2}}{\sum_{i=1}^{n}{\left(y_{i}-\bar{y}\right)}^{2}},$$

(3) $$Accuracy=\frac{Number\;of\;correct\;predictions}{Total\;number\;of\;predictions},$$

where $y_{i}$ is the ground truth of the leaf-counting number for each image, ${\hat{y}}_{i}$ is the predicted value without rounding for the same image, *n* is the total number of images in the dataset, and $\bar{y}$ is the average value of the leaf-counting number without rounding over all images.

#### 2.8.2. Qualitative Evaluation Metrics

We are also interested in investigating the inner mechanism of our regression process. To this end, we employ neural network visualization techniques to qualitatively convey the features and explore the feature maps based on regression results. These visualization techniques can broadly be separated into three categories: input modification methods [46], backpropagation-based methods [47], and class activation mapping (CAM) methods [48]. We use the input modification method and the gradient-weighted class activation mapping (Grad-CAM) process [49] to detect significant features and their corresponding spatial positions that remarkably contribute to the regression results from different datasets.

**Figure 5:**
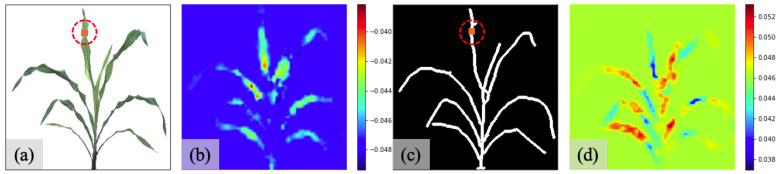
Heatmaps from input modification method for datasets S1: (**a**) original sorghum image with a sliding window of size (10×10) (within the red dash circle); (**b**) heatmap derived from Figure 5a; (**c**) skeleton image of Figure 5a with a sliding window of size (10×10) (within the red dash circle); (**d**) heatmap derived from Figure 5c.

Dobrescu et al. [42,50] used the input modification method by imposing a black sliding window (60×60) into an input image and making it traverse the image. The introduced sliding window can influence the original prediction without disturbance. Based on the difference between ground truth and prediction at each window position, the significant regions that have remarkable performance influences can be detected. We use the same process in our work and create a white sliding window for the original sorghum image datasets and a black sliding window for the skeleton image datasets. The sliding window colors are determined by the background colors for each dataset. The size of the sliding box is set to be 10×10, and this is the approximate width for an individual leaf in our case. The red circles in Figure 5a,c illustrate the size and shape of the sliding boxes related to leaves. When traversing the entire image, the amount of movement for each sliding box is set to be 5.

To further derive the feature map and explain the inner mechanism of the models, we apply Grad-CAM (gradient weighted class activation mapping) [49] to the regression outputs. The Grad-CAM mechanism can be generalized in that the gradients of the targets flow into the final convolutional layer, and then a coarse localization map is produced based on the flow intensities.

## 3. Results

We first introduce the experiment dataset configurations in Section 3.1. Then, we present the performance analysis on our datasets and the corresponding comparison with the state-of-the-art methods in Section 3.2 and Section 3.3 with respect to different experiment dataset configurations.

### 3.1. Experiment Dataset Configuration

We first employ the sorghum plant images to train, validate, and test the models and then use the maize plant images to further investigate the ability of our modified Inception-ResNet-V2 to predict the leaf numbers on different monocot species. To gain a deeper understanding of the performance of our methods, we divide the image datasets of sorghum and maize plants into two types, one with all separated leaves and the other with both separated leaves and occluded leaves.

#### 3.1.1. Sorghum Dataset Configuration

**Figure 6:**
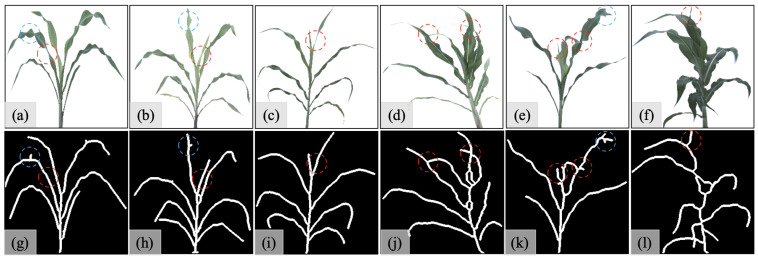
Dataset description: (**a**–**c**) are examples of original sorghum images in dataset S1O; (**d**–**f**) are examples of original sorghum images in dataset S2O; (**g**–**i**) correspond skeleton structure images S1S for (**a**–**c**); (**j**–**l**) correspond skeleton structure images S2S for (**d**–**f)**.

The leaf-separated sorghum dataset (referred to as S1O) contains 1000 sorghum images with clearly-separated leaves, and the other mixed sorghum dataset (referred to as S2O) contains 500 sorghum images with clearly separated leaves (randomly selected from S1O) and 500 sorghum images with occluded leaves. We then generate skeleton-structure datasets S1S and S2S, respectively, from S1O and S2O, as shown in Figure 6.

Figure 6a–c give the examples of original sorghum images in the dataset S1O, and Figure 6g–i show the corresponding skeleton structure images in S1S. The examples prove that the skeleton structure can strengthen the weak connections in the leaves. The leaf segments in the red dashed circles in Figure 6a–c show the weak segments in the original sorghum images. Compared to those parts, the same leaf segments in the red dashed circles in Figure 6g–i present the same widths as the surrounding leaves, which indicates that the skeleton structure can eliminate the weak connection effects in original sorghum images. However, the skeleton structure also has its shortcomings. Under conditions such as the high variations of width in one leaf, the skeleton structure can bring unexpected noises to skeleton-structured leaves, as indicated in the blue dashed circles in Figure 6g,h. These new noises have the same appearance as the actual small leaf tips that appeared in the skeleton-structured form. We can see that there are no extra leaves inside the blue dashed circles from the original images (Figure 6a,b). However, the extra noises that appeared as tips are shown in the skeleton structures (e.g., the blue dashed circles in Figure 6g,h), which conflicts with reality.

Figure 6d–f give the examples of original sorghum images in S2O, and Figure 6j–l show the corresponding skeleton structure images in S2S. This dataset is used to investigate the model’s performance on the sorghum images with occluded leaves. The leaves in the red dashed circles show the variations of leaves’ occlusion conditions. The red dashed circles in Figure 6d indicate the appearances of the leaves with a hidden tip (the left circle) and the occluded leaves (the right circle) in the sorghum images. The red dashed circles in Figure 6e,f show the appearances of small leaves shown in the sorghum images. From the red dashed circles in Figure 6k,l, we can see that their shapes can appear as the natural tips on the skeleton-structured leaves. Moreover, Figure 6f also suggests that our model intends to solve the counting problems when the sorghum plants do not present the best views in the images. The blue dashed circle in Figure 6e illustrates the same noises introduced in the dataset S2O, which could be recognized as the extra tips due to the skeleton structures.

As discussed in Section 2.5, there are twelve augmentation methods, among which six generate realistic synthetic images, and the other six generate non-realistic ones, as shown in the first and second rows of Figure 4, respectively. We generate the datasets S1O_6A, S1S_6A, S2O_6A, and S2S_6A by applying the first six methods to S1O, S1S, S2O, and S2S, respectively, in order to study the effect of realistic augmentation methods on the model performance. To further investigate the performance difference between the realistic and non-realistic synthetic images, we also apply all the twelve augmentation methods to S2O and S2S, and then create the datasets S2O_12A and S2S_12A, respectively. Therefore, there are eight sorghum datasets in our experiments.

For the datasets without augmentation methods (S1O, S1S, S2O, and S2S), there are 1000 images with 800 training samples (80%), 100 validation samples (10%), and 100 testing samples (10%) in each dataset. The augmentation methods are only applied to the training datasets to enhance their size and variety and, thereby, the quality of trained models. The validation datasets and the testing datasets remain the same. We first apply the six realistic augmentation methods and extend the training datasets and have 4800 training samples. Meanwhile, we still have 100 validation samples and 100 testing samples, resulting in a total of 5000 images in the dataset. Second, we apply all twelve methods to generate realistic and non-realistic synthetic images and extend the training dataset to have 9600 training samples, thereby having 9800 images by including 100 validation samples and 100 testing samples. Therefore, in the augmented datasets S1O_6A, S1S_6A, S2O_6A, and S2S_6A, there are 5000 images with 4800 training samples (96%), 100 validation samples (2%), and 100 testing samples (2%) in each dataset. For the augmented datasets S2O_12A and S2S_12A, there are 9800 images with 9600 training samples (97.96%), 100 validation samples (1.02%), and 100 testing samples (1.02%) in each dataset. We use S1 to refer to the leaf-separated sorghum dataset S1O and its derived datasets, and otherwise S2.

#### 3.1.2. Maize Dataset Configuration

**Figure 7:**
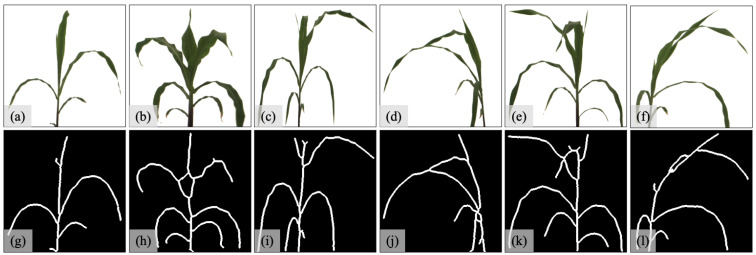
Dataset description: (**a**–**c**) are examples of original maize images in dataset M1O; (**d**–**f**) are examples of original maize images in dataset M2O; (**g**–**i**) correspond skeleton structure images for (**a**–**c**); and (**j**–**l**) correspond skeleton structure images for (**d**–**f**).

The maize images are only used to evaluate the model performance on general monocot plants. Thus, we generate four different datasets, each containing 100 maize images, as shown in Figure 7. The dataset M1O is the original maize dataset containing 100 maize images with clearly separated leaves (Figure 7a–c). The dataset M1S is the skeleton-structured maize dataset corresponding to M1O (Figure 7g–i). The dataset M2O is the original maize dataset containing 100 maize images with 50 clearly separated-leaf images randomly selected from M1O and 50 occluded-leaf images (Figure 7d–f). The dataset M2S is the skeleton-structured maize dataset corresponding to M2O (Figure 7j–l). We use M1 to refer to the leaf-separated maize dataset M1O and its derived datasets, and otherwise M2.

### 3.2. Results from Sorghum Datasets S1 and Maize Datasets M1

The sorghum datasets S1 are used to train the model and predict the number of leaves in the images without occluded conditions. Specifically, S1O, S1S, S1O_6A, and S1S_6A are trained and evaluated at 500 epochs. We also employ the maize datasets M1O and M1S to directly evaluate the best model trained from the sorghum datasets.

We employ RMSE, $R^{2}$, and accuracy to evaluate the performance of the sorghum and maize datasets, and compare the resulting performance with the existing works. Table 2 shows the evaluation metrics for each dataset. We can easily see that the RMSE values of our approach from all experiments based on dataset S1 are less than 0.20, which indicates that the difference between the ground truth and the prediction is significantly smaller than half of a leaf (0.5). The $R^{2}$ values from all experiments are above 0.98, and this suggests that our model can explain at least 98% variability of the leaf-counting numbers around their mean. The testing accuracy from most experiments is 99%, which further indicates the excellent performance of all experiments. These three evaluation metrics show that this model can generate an accurate count of leaves and present an outstanding performance from all experiments when there are no occlusions in sorghum datasets.

We also compare the evaluation metrics among different datasets. Due to the excellent performance of the original sorghum datasets S1O and S1O_6A, the skeleton-structured datasets S1S and S1S_6A cannot remarkably improve the model performance. Moreover, the results indicate the significant role of augmentation methods in improving the model performance. These argumentation methods improve the RMSE value from 0.17 to 0.10 for both S1O_6A and S1S_6A. In addition, the $R^{2}$ values are also increased, which is resulting from the augmentation. Because the performances among all experiments are close, it is hard to see the advantages of the skeleton structures and augmentation methods. We will evaluate those two techniques further in Section 3.3.

To investigate the performance of the best model on maize plants, we apply the trained model from S1O_6A to M1O, and the trained model from S1S_6A to M1S, respectively. From Table 2, we can see that for predicted maize leaf numbers, the RMSE values are less than 0.4, the $R^{2}$ values are larger than 0.85, and all testing accuracy is above 90%. These results indicate the models trained from the sorghum datasets can moderately predict the number of maize leaves. It can also be concluded that skeleton-structure images can reduce the shape difference resulting from different species and improve the prediction performance for monocot plants.

**Figure 8:**
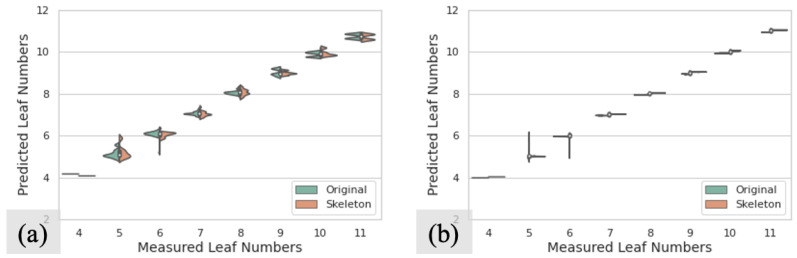
The distributions of predicted leaf numbers in testing datasets without occlusion: (**a**) prediction distributions in S1 datasets without augmentation; (**b**) prediction distributions in S1 datasets with six transformations. In each plot, green parts represent the results from the original images, and orange parts represent the results from skeleton images.

Figure 8 shows the distributions of predicted values in different sorghum testing datasets without occlusions. Figure 8a,b present the sorghum datasets without augmentations and with six transformations, respectively. For each plot in Figure 8, the horizontal axis represents the measured different numbers of leaves from the ground truth, and the vertical axis shows the distribution of quantitative data from the prediction for each measured leaf number. Each violin is set to have the same area. The green parts represent the results from the original images, and the orange parts represent the results from skeleton images. It can be seen that the shapes for green and orange parts are similar over the same ground truths, which means that there is no significant difference between the resulting distributions from original images and skeleton images, which is consistent with the RMSE results as shown in Table 2. We can see that the augmentation methods can considerably improve the performance by concentrating the predicted values on ground truth values.

These results are also compared with the existing works conducted on a similar dataset. The method Leaf-count-net+FV [6] uses a dataset with 2845 maize images and produces a relatively high RMSE of 0.5. Miao et al. [9] propose several methods trained with an enormous dataset, all of which obtain poor performance with an RMSE larger than 1 and a testing accuracy of less than 60%. Compared to them, our experiments show superior performance.

Qualitative evaluation: To qualitatively assess the model performance, we employ the input modification method and Grad-CAM method to visualize the feature maps and detect significant spatial positions from the images.

Figure 5 includes the heatmaps from experiments on the datasets S1O_6A and S1S_6A, as shown in Figure 5b,d. They directly show the difference between the ground truth number of leaves and the predicted number of leaves when one block is located in each position. In our colormap, when the color turns to be cooler, a relatively more minor predicted value is presented; when the color becomes warmer, a relatively larger predicted value is given. The ranges for the difference are 0.008 in Figure 5b and 0.014 in Figure 5d, both of which are negligible compared to one whole leaf. We can also see that the disconnected leaves cut by the sliding box will increase the predicted value. It means the regression output is primarily determined by the number of separated leaves instead of areas. In addition, the heatmaps generally highlight leaves, corresponding to the positions that most influence the regression results, rather than stems. It also shows that if the leaves are disconnected from the root of the leaf, it will generally increase the regression number of the leaves. If the leaves are disconnected from the tip of the leaf, it will naturally decrease the regression number of the leaves.

**Figure 9:**
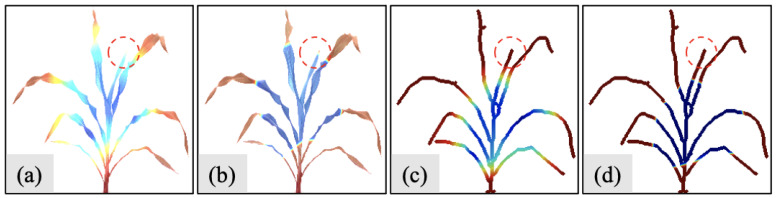
Heatmaps from Grad-CAM method for datasets S1: (**a**) heatmap derived from Figure 5a; (**b**) emphasized heatmap derived from Figure 5a; (**c**) heatmap derived from Figure 5c; (**d**) emphasized heatmap derived from Figure 5c.

Figure 9 shows the heatmaps from the Grad-CAM method for S1 datasets. We use the same colormap to identify significant locations, where the red color labels the areas that contribute most to the results, and the blue regions indicate the less contributed areas. We also apply the sigmoid function to the original heatmaps to distinguish the boundary between these two regions.

Figure 9a,c are the original Grad-CAM heatmaps derived from the sorghum image in Figure 5a and the skeleton image in Figure 5c. Figure 9b,d are the emphasized heatmaps after applying the sigmoid operation to Figure 9a,c. We can see that all heatmaps highlight the tips of leaves, which suggests the leaf tips play a significant role in determining the number of leaves during the regression process. We also annotate the same leaf in the red circles in all heatmaps. Compared to the heatmaps from the original sorghum image, the heatmaps from the skeleton image present a significant tip effect that contributes to the regression result.

### 3.3. Results from Sorghum Datasets S2 and Maize Datasets M2

Experiment descriptions: The sorghum datasets S2 are used to train the model and predict the number of leaves in the images with occluded conditions. Specifically, S2O, S2S, S2O_6A, S2S_6A, S2O_12A, and S2S_12A are trained and evaluated at 500 epochs. We also employ the maize datasets M2O and M2S to directly evaluate the best model trained from the sorghum datasets S2.

Quantitative evaluation: Following the same quantitative evaluations, the experiments are evaluated by the metrics of RMSE, $R^{2}$, and accuracy. We compare these performances with the work of [9] that also employ the natural maize and sorghum images to count the number of leaves by using regression CNNs and Faster R-CNNs.

Table 3 shows the evaluation results for the datasets of S2 and M2. From Table 3, we can see that the modified Inception-ResNet-V2 architecture can accurately predict sorghum leaves number when there are occluded leaves. The RMSE values from the S2 datasets are less than half a leaf (<0.5), $R^{2}$ are above 0.90, and the testing accuracy is more significant than 75%. In addition, we can see that the experiments trained with skeleton structure images can achieve better performance than those from original sorghum images. Both datasets S2S and S2S_6A present a lower RMSE and a higher $R^{2}$ when they are compared with datasets S2O and S2O_6A. Specifically, S2S_6A improves the testing accuracy from 85% to 88%. S2S_12A also improves the model performance by lowering RMSE, improving both $R^{2}$ and testing accuracy, when it is compared to S2O_12A. In addition, we can easily see that augmentations can help models improve their performance. Compared to the datasets without augmentation methods, the augmented datasets show a better performance. Compared to the performance from S2O, S2O_6A decreases the RMSE value from 0.48 to 0.36, improves the $R^{2}$ value from 0.94 to 0.96, and increases the testing accuracy by approximately 8%. S2S_6A also decreases the RMSE value by 0.12, improves $R^{2}$ to 0.97, and increases the testing accuracy to 88%. We also investigate the effect of unrealized augmentations on sorghum images. We can see that the unrealized augmentations can significantly improve the models’ performance. Compared to S2O_6A, S2O_12A increases the testing accuracy from 85% to 87%. Compared to S2S_6A, S2S_12A increases the testing accuracy from 88% to 91%.

The parameters trained from S2O_12A and S2S_12A are applied to the maize image datasets M2O and M2A. It can be seen that the maize testing datasets present a less optimal performance compared to the sorghum testing datasets. This may result from the different occluded conditions of maize plants and sorghum plants and the different typologies between the two species. However, our approach still outperforms the existing method on maize, as shown in Table 3.

**Figure 10:**
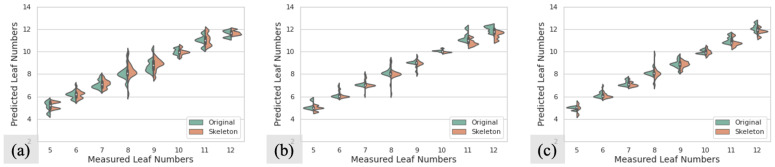
The distributions of predicted leaf numbers in testing datasets with occlusion: (**a**) prediction distributions in S2 datasets without augmentation; (**b**) prediction distributions in S2 datasets with six transformations; (**c**) prediction distributions in S2 datasets with twelve transformations. In each plot, green parts represent the results from the original images, and orange parts represent the results from skeleton images.

Figure 10 shows the distributions of predicted values in different testing datasets with occlusions. Figure 10a–c present the distributions for sorghum datasets without augmentations, with six transformations, and with twelve transformations, respectively. It can be seen that, compared to the original images, the results from the skeleton images are closer to the corresponding ground truth values. Furthermore with the number of transformations increasing, more predicted values locate around the ground truth values.

Miao et al. [9] presented an RMSE value of 1.28 and an accuracy of 33% when they trained the regression CNN on maize dataset, an RMSE value of 1.06 and an accuracy of 39% when they tested the regression CNN on sorghum dataset, and an RMSE value of 1.33 and an accuracy of 43% while using faster-RCNN on the maize dataset. Compared with their work, our model largely reduces the RMSE from more than one leaf (>1) to half of a leaf (<0.5) and improves the accuracy. We can see that all evaluation metrics from our experiments outperform their results. All results show that our method does not only improve the accuracy of leaf-counting even with overlaps and occlusions, but also lower the training cost with fewer annotations, compared to the previous state-of-the-art approaches.

**Figure 11:**
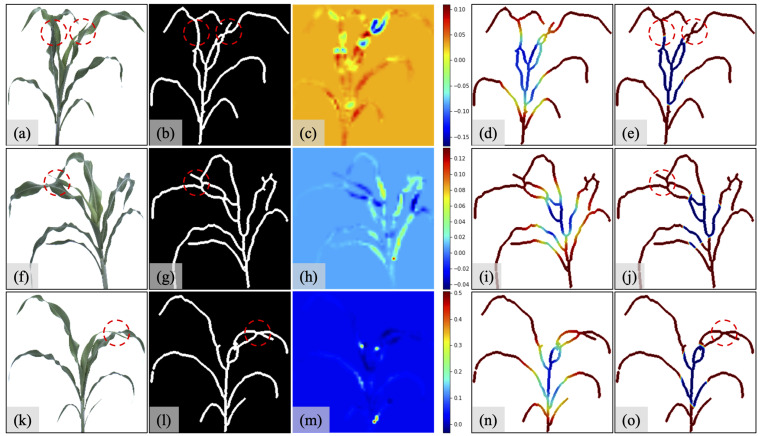
Qualitative evaluations on datasets S2. The first row shows the qualitative analysis of dataset S2S, the second row shows the qualitative analysis of dataset S2S_6A, and the third row shows the qualitative analysis of dataset S2S_12A. In each row, the first column (**a**,**f**,**k**) shows original sorghum images, the second column (**b**,**g**,**l**) shows skeleton structure images corresponding to the first column, the third column (**c**,**h**,**m**) shows heatmaps from input modification method for second column images as input, fourth column (**d**,**i**,**n**) shows heatmaps from Grad-CAM for second column images as input. The fifth column (**e**,**j**,**o**) shows emphasized heatmaps from Grad-CAM for second-column images as input.

Qualitative evaluation: To investigate the effect of different augmentation methods, we apply the input modification method and Grad-CAM to the experiments on the datasets S2S, S2S_6A, and S2S_12A, as shown in Figure 11. The first to third rows show the qualitative analysis results of S2S, S2S_6A, and S2S_12A, respectively.

From the first column in Figure 11, we can see that the modified Inception-ResNet-V2 can predict the number of sorghum leaves with occlusion, as indicated in the red circles in Figure 11a,f,k. The third column highlights the significant positions for regression using the input modification method. The color bars show the differences between the predicted value and ground truths, with a range of 0.25, 0.16, and 0.5, which are less than half of a leaf. We can see that the occlusion region between two leaves largely influences the regression results. When the sliding windows cover the occlusions, the predicted values are reduced and indicated by the darkest areas in Figure 11c,h,m. The fourth and fifth columns show the heatmap analysis from Grad-CAM. The fourth column indicates leaves are the most critical features for leaf-counting regression. They present a much darker red color than the stems. After the sigmoid operation, the fifth column shows that the detection is focused on the tips of the leaves, and the methods can detect the leaves when there are occluded leaves (within the red circles in Figure 11e,j,o).

## 4. Conclusions

In this paper, we present a modified Inception-ResNet-V2 architecture to predict leaf numbers in monocot plants and investigate the effect of skeleton structure and augmentation methods on model performance. We employ sorghum plant images to train, validate, and test the models, and then use the maize plant images to further investigate the ability of the model to predict the leaf numbers on different monocot species. For each species, we create two types of datasets: one dataset containing images with clearly separated leaves, and the other dataset containing images with both clearly separated leaves and occluded leaves. We also apply the skeleton structure to all original datasets and augment the training datasets by using geometric transformations.

There are two kinds of noises appearing in our model: one from the environments such as the illumination pixels and equipment pixels, and the other from the “extra tips” after skeletonization. The environmental noises are considered small independent components from the main plant. We empirically set a threshold and remove the noises that are smaller than the threshold during the image preprocessing. For the noises introduced by skeletonization, the results show the robustness of the model against the noise effect. The experiments from dataset S1 and dataset M1 achieve relatively lower RMSE and higher accuracy when there are “extra tips” noises shown in the datasets.

We evaluate our experiments from quantitative metrics and qualitative visualization. The experiments on the datasets S1 present the best performance with an RMSE of 0.10, a $R^{2}$ value of 0.99, and a testing accuracy of 0.99 for sorghum images with clearly separated leaves. The experiments on the dataset S2 achieve the best performance when the model is trained with the skeleton structured dataset augmented by all transformations (i.e., S2S_12A). It achieves the performance with an RMSE of 0.33, a $R^{2}$ value of 0.97, and a testing accuracy of 0.91. We also apply the best models to maize datasets to evaluate the models. The best performance is achieved with an accuracy of 91% for clearly separated maize leaves and 73% for occluded maize leaves. The comparison results show our approach outperforms the previous state-of-the-art work. Our experiments indicate that both the skeleton-structured images and the augmentation methods can promise better results. Even the non-realistic synthetic image is able to help detect the leaf tip feature and improve the model performance.

We also compare our results with the existing approaches conducted on a similar dataset. The method Leaf-count-net+FV [6] uses a dataset with 2845 maize images and four levels of labels according to the number of leaves. They produce a relatively high RMSE of 0.5 with an absolute difference of 0.35. Miao et al. [9] propose both regression models and object detection models on an enormous dataset with more than 150,000 images, including both clearly separated leaves and occluded leaves. For experiments on clearly separated leaves, the best performance from their work achieves an RMSE of 0.96 and a testing accuracy of 56%. For experiments on occluded leaves, the best performance from their work gets an RMSE of 1.06 and a testing accuracy of 43%. Compared to their results, all our experiments achieve a significantly lower RMSE and higher accuracy by using fewer images with 1000 original images in the dataset, which shows the superior performance of our methods.

Through careful analysis with the input modification method and the Grad-CAM method, we have demonstrated that our model is able to generate the feature maps and detect the significant parts for regression predictions of the leaves counting numbers. Our method also suggests that the leaf tips make the most significant contribution to the regression results.

While skeletonization has significantly increased the performance of the neural work, we note that there are still subtle noises presented in resulting skeletons due to non-trivial plant structures in our current work. In the future, we plan to derive a more accurate skeleton structure, mitigating extra noises and helping us to solve more image recognition problems in monocot plant images, such as semantic segmentation and object detection for the leaves and stems. We aim to gain a deeper understanding of the relationship between skeleton structures and regression results, which may help us identify other possible abstracts or features that can be extracted from plant images and further improve the model performance. We also plan to explore more regression- and segmentation-based methods and compare their performance in leaf-counting for monocot plants. In particular, we will exploit the advantages of these existing approaches and develop new DNN architectures to address the occlusion problem in a scalable manner.

## Figures and Tables

**Table 1:** Hyper-parameters used for training.

Batch Size	Learning Rate	Optimizer	Loss Function
16	0.0001	RMSprop	Mean Squared Error

**Table 2:** Performance comparison between our and existing methods on S1 and M1.

Dataset & Method	RMSE	$R^{2}$	Accuracy
S1O (ours) ${}^{a}$	0.17	0.98	0.99
S1S (ours) ${}^{a}$	0.17	0.99	0.99
S1O_6A (ours) ${}^{a}$	0.10	0.99	0.99
S1S_6A (ours) ${}^{a}$	0.10	0.99	0.99
M1O (ours) ${}^{b}$	0.35	0.86	0.90
M1S (ours) ${}^{c}$	0.29	0.91	0.91
(Leaf-count-net+FV) ([6]) ${}^{a}$	0.57	-	-
Regression-CNN ([9]) ${}^{a}$	0.96	0.87	0.45
Regression-CNN ([9]) ${}^{d}$	1.06	0.64	0.39
Faster-RCNN ([9]) ${}^{a}$	1.00	0.88	0.56

S1O: The original sorghum dataset contains 1000 sorghum images with clearly separated leaves. S1S: The generated skeleton-structure sorghum dataset corresponding to S1O. S1O_6A: The generated augmented dataset by applying six augmentation methods in the first row of Figure 4 to S1O. S1S_6A: The generated skeleton-structure sorghum dataset corresponding to S1O_6A. M1O: The original maize dataset contains 100 maize images with clearly separated leaves. M1S: The generated skeleton-structure maize dataset corresponding to M1O. *^a^* Model is trained and tested on the sorghum dataset. *^b^* Model is trained on S1O_6A and tested on M1O. *^c^* Model is trained on S1S_6A and tested on M1S. *^d^* Model is trained on the maize dataset and tested on the sorghum dataset.

**Table 3:** Performance comparison between our and existing methods on M2 and S2.

Dataset & Method	RMSE	$R^{2}$	Accuracy
S2O (ours) ${}^{a}$	0.48	0.94	0.77
S2S (ours) ${}^{a}$	0.46	0.94	0.77
S2O_6A (ours) ${}^{a}$	0.36	0.96	0.85
S2S_6A (ours) ${}^{a}$	0.34	0.97	0.88
S2O_12A (ours) ${}^{a}$	0.35	0.97	0.87
S2S_12A (ours) ${}^{a}$	0.33	0.97	0.91
M2O (ours) ${}^{b}$	0.56	0.63	0.65
M2S (ours) ${}^{c}$	0.51	0.68	0.73
Regression-CNN ([9]) ${}^{a}$	1.28	0.79	0.33
Regression-CNN ([9]) ${}^{d}$	1.06	0.64	0.39
Faster-RCNN ([9]) ${}^{a}$	1.33	0.78	0.43

S2O: The original sorghum dataset contains 500 sorghum images with clearly separated leaves (randomly selected from S1O) and 500 sorghum images with occluded leaves. S2S: The generated skeleton-structure sorghum dataset corresponding to S2O. S2O_6A: The generated augmented dataset by applying six augmentation methods in the first row of Figure 4 to S2O. S2S_6A: The generated skeleton-structure sorghum dataset corresponding to S2O_6A. S2O_12A: The generated augmented dataset by applying all transformations in Figure 4 to S2O. S2S_12A: The generated skeleton-structure sorghum dataset corresponding to S2O_12A. M2O: The original maize dataset contains 500 maize images with clearly separated leaves (randomly selected from M1O) and 500 maize images with occluded leaves. M2S: The generated skeleton-structure maize dataset corresponding to M2O. *^a^* Model is trained and tested on the sorghum dataset. *^b^* Model is trained on S1O_6A and tested on M1O. *^c^* Model is trained on S1S_6A and tested on M1S. *^d^* Model is trained on the maize dataset and tested on the sorghum dataset.

## Data Availability

Not applicable.

## References

[1] Farjon G., Itzhaky Y., Khoroshevsky F., Bar-Hillel A. (2021). Leaf counting: Fusing network components for improved accuracy. Front. Plant Sci..

[2] Buzzy M., Thesma V., Davoodi M., Mohammadpour Velni J. (2020). Real-time plant leaf counting using deep object detection networks. Sensors.

[3] Praveen Kumar J., Domnic S. (2020). Rosette plant segmentation with leaf count using orthogonal transform and deep convolutional neural network. Mach. Vis. Appl..

[4] Minervini M., Fischbach A., Scharr H., Tsaftaris S. (2015). Plant Phenotyping Datasets. http://www.Plant-phenotyping.org/datasets.

[5] Minervini M., Fischbach A., Scharr H., Tsaftaris S.A. (2016). Finely-grained annotated datasets for image-based plant phenotyping. Pattern Recognit. Lett..

[6] Jiang B., Wang P., Zhuang S., Li M., Li Z., Gong Z. (2019). Leaf counting with multi-scale convolutional neural network features and fisher vector coding. Symmetry.

[7] Szegedy C., Vanhoucke V., Ioffe S., Shlens J., Wojna Z. Rethinking the inception architecture for computer vision. Proceedings of the IEEE Conference on Computer Vision and Pattern Recognition.

[8] Cui S.L., Tian F. (2009). Face recognition method based on SIFT feature and fisher. Comput. Eng..

[9] Miao C., Guo A., Thompson A.M., Yang J., Ge Y., Schnable J.C. (2021). Automation of leaf counting in maize and sorghum using deep learning. Plant Phenome J..

[10] He K., Zhang X., Ren S., Sun J. Deep residual learning for image recognition. Proceedings of the IEEE Conference on Computer Vision and Pattern Recognition.

[11] Ren S., He K., Girshick R., Sun J. Faster r-cnn: Towards real-time object detection with region proposal networks. Proceedings of the Advances in Neural Information Processing Systems, Palais des Congrès de Montréal.

[12] Xiang L., Bao Y., Tang L., Ortiz D., Salas-Fernandez M.G. (2019). Automated morphological traits extraction for sorghum plants via 3D point cloud data analysis. Comput. Electron. Agric..

[13] Szegedy C., Ioffe S., Vanhoucke V., Alemi A.A. Inception-v4, inception-resnet and the impact of residual connections on learning. Proceedings of the Thirty-First AAAI Conference on Artificial Intelligence.

[14] Gaillard M., Miao C., Schnable J., Benes B. (2020). Sorghum segmentation by skeleton extraction. Proceedings of the European Conference on Computer Vision.

[15] Abed S.H., Al-Waisy A.S., Mohammed H.J., Al-Fahdawi S. (2021). A modern deep learning framework in robot vision for automated bean leaves diseases detection. Int. J. Intell. Robot. Appl..

[16] Zhang C., Zhou P., Li C., Liu L. A convolutional neural network for leaves recognition using data augmentation. Proceedings of the 2015 IEEE International Conference on Computer and Information Technology; Ubiquitous Computing and Communications; Dependable, Autonomic and Secure Computing; Pervasive Intelligence and Computing.

[17] Montavon G., Samek W., Müller K.R. (2018). Methods for interpreting and understanding deep neural networks. Digit. Signal Process..

[18] Yu Y., Liang S., Samali B., Nguyen T.N., Zhai C., Li J., Xie X. (2022). Torsional capacity evaluation of RC beams using an improved bird swarm algorithm optimised 2D convolutional neural network. Eng. Struct..

[19] Kaliyar R.K., Goswami A., Narang P., Sinha S. (2020). FNDNet—A deep convolutional neural network for fake news detection. Cogn. Syst. Res..

[20] Mo H., Chen B., Luo W. Fake faces identification via convolutional neural network. Proceedings of the 6th ACM Workshop on Information Hiding and Multimedia Security.

[21] Kang K., Li H., Yan J., Zeng X., Yang B., Xiao T., Zhang C., Wang Z., Wang R., Wang X. (2017). T-cnn: Tubelets with convolutional neural networks for object detection from videos. IEEE Trans. Circuits Syst. Video Technol..

[22] Strubell E., Ganesh A., McCallum A. (2019). Energy and policy considerations for deep learning in NLP. arXiv.

[23] Pouyanfar S., Chen S.C., Shyu M.L. An efficient deep residual-inception network for multimedia classification. Proceedings of the 2017 IEEE International Conference on Multimedia and Expo (ICME).

[24] Yang S., Lin G., Jiang Q., Lin W. (2019). A dilated inception network for visual saliency prediction. IEEE Trans. Multimed..

[25] Das D., Santosh K., Pal U. (2020). Truncated inception net: COVID-19 outbreak screening using chest X-rays. Phys. Eng. Sci. Med..

[26] Alom M.Z., Yakopcic C., Nasrin M.S., Taha T.M., Asari V.K. (2019). Breast cancer classification from histopathological images with inception recurrent residual convolutional neural network. J. Digit. Imaging.

[27] Yu Y., Samali B., Rashidi M., Mohammadi M., Nguyen T.N., Zhang G. (2022). Vision-based concrete crack detection using a hybrid framework considering noise effect. J. Build. Eng..

[28] Farooq M., Hafeez A. (2020). Covid-resnet: A deep learning framework for screening of covid19 from radiographs. arXiv.

[29] Russakovsky O., Deng J., Su H., Krause J., Satheesh S., Ma S., Huang Z., Karpathy A., Khosla A., Bernstein M. (2015). Imagenet large scale visual recognition challenge. Int. J. Comput. Vis..

[30] Da Silva N.B., Gonçalves W.N. Regression in Convolutional Neural Networks applied to Plant Leaf Counting. Proceedings of the Anais do XV Workshop de Visão Computacional. SBC.

[31] Krishnamoorthy N., Prasad L.N., Kumar C.P., Subedi B., Abraha H.B., Sathishkumar V. (2021). Rice leaf diseases prediction using deep neural networks with transfer learning. Environ. Res..

[32] Du S., Lindenbergh R., Ledoux H., Stoter J., Nan L. (2019). AdTree: Accurate, detailed, and automatic modelling of laser-scanned trees. Remote Sens..

[33] Guo Z., Hall R.W. (1989). Parallel thinning with two-subiteration algorithms. Commun. ACM.

[34] Ajiboye A., Abdullah-Arshah R., Qin H., Isah-Kebbe H. (2015). Evaluating the effect of dataset size on predictive model using supervised learning technique. Int. J. Comput. Syst. Softw. Eng..

[35] Taylor L., Nitschke G. Improving deep learning with generic data augmentation. Proceedings of the 2018 IEEE Symposium Series on Computational Intelligence (SSCI).

[36] Krizhevsky A., Sutskever I., Hinton G.E. Imagenet classification with deep convolutional neural networks. Proceedings of the Advances in Neural Information Processing Systems.

[37] Deng J., Dong W., Socher R., Li L.J., Li K., Fei-Fei L. Imagenet: A large-scale hierarchical image database. Proceedings of the 2009 IEEE Conference on Computer Vision and Pattern Recognition.

[38] Shorten C., Khoshgoftaar T.M. (2019). A survey on image data augmentation for deep learning. J. Big Data.

[39] Kuznichov D., Zvirin A., Honen Y., Kimmel R. Data augmentation for leaf segmentation and counting tasks in rosette plants. Proceedings of the IEEE/CVF Conference on Computer Vision and Pattern Recognition Workshops.

[40] Ge Y., Bai G., Stoerger V., Schnable J.C. (2016). Temporal dynamics of maize plant growth, water use, and leaf water content using automated high throughput RGB and hyperspectral imaging. Comput. Electron. Agric..

[41] Vanderlip R. (1979). How a Sorghum Plant Develops.

[42] Dobrescu A., Valerio Giuffrida M., Tsaftaris S.A. Leveraging multiple datasets for deep leaf counting. Proceedings of the IEEE International Conference on Computer Vision Workshops.

[43] Itzhaky Y., Farjon G., Khoroshevsky F., Shpigler A., Bar-Hillel A. Leaf counting: Multiple scale regression and detection using deep CNNs. Proceedings of the BMVC.

[44] Minervini M., Abdelsamea M.M., Tsaftaris S.A. (2014). Image-based plant phenotyping with incremental learning and active contours. Ecol. Inform..

[45] Scharr H., Minervini M., French A.P., Klukas C., Kramer D.M., Liu X., Luengo I., Pape J.M., Polder G., Vukadinovic D. (2016). Leaf segmentation in plant phenotyping: A collation study. Mach. Vis. Appl..

[46] Zeiler M.D., Fergus R. (2014). Visualizing and understanding convolutional networks. Proceedings of the European Conference on Computer Vision.

[47] Dobrescu A., Valerio Giuffrida M., Tsaftaris S.A. Understanding deep neural networks for regression in leaf counting. Proceedings of the IEEE/CVF Conference on Computer Vision and Pattern Recognition Workshops.

[48] Zhou B., Khosla A., Lapedriza A., Oliva A., Torralba A. Learning deep features for discriminative localization. Proceedings of the IEEE Conference on Computer Vision and Pattern Recognition.

[49] Selvaraju R.R., Cogswell M., Das A., Vedantam R., Parikh D., Batra D. Grad-cam: Visual explanations from deep networks via gradient-based localization. Proceedings of the IEEE International Conference on Computer Vision.

[50] Dobrescu A., Giuffrida M.V., Tsaftaris S.A. (2020). Doing more with less: A multitask deep learning approach in plant phenotyping. Front. Plant Sci..

